# Epilepsy care pathway: The Finnish model

**DOI:** 10.1002/epi4.13093

**Published:** 2024-11-09

**Authors:** Reetta Kälviäinen, Zakarya Hadj‐Allal, Jarkko Kirjavainen, Reina Roivainen, Tarja Linnankivi, Jukka Peltola, Kai Eriksson, Salla Lamusuo, Tuire Lähdesmäki, Johanna Annunen, Päivi Vieira, Virpi Tarkiainen, Leena Jutila, Anni Saarela, Leena Kämppi, Liisa Metsähonkala, Eija Gaily, Niina Lähde, Jaana Antinmaa, Sini Erme, Anna‐Leena Pirttisalo, Jari Virolainen, Milla Ylijoki, Laura Kela, Jonna Komulainen‐Ebrahim, Paula Sorjonen, Finnish Severe Epilepsy Coordination Group

**Affiliations:** ^1^ Kuopio Epilepsy Center, Neurocenter Kuopio University Hospital Kuopio Finland; ^2^ Institute of Clinical Medicine, School of Medicine, Faculty of Health Sciences University of Eastern Finland Kuopio Finland; ^3^ Kuopio Epilepsy Center, Pediatric Neurology Kuopio University Hospital Kuopio Finland; ^4^ Department of Neurology, Epilepsia Helsinki Helsinki University Hospital Helsinki Finland; ^5^ Department of Pediatric Neurology, Epilepsia Helsinki Helsinki University Hospital Helsinki Finland; ^6^ Department of Neurology Tampere University Hospital Tampere Finland; ^7^ Department of Neurology Tampere University Tampere Finland; ^8^ Department of Pediatric Neurology Tampere University Hospital Tampere Finland; ^9^ Tampere Center for Child, Adolescent and Maternal Health Research (TamCAM) Tampere University Tampere Finland; ^10^ Department of Neurology Turku University Hospital Turku Finland; ^11^ Department of Pediatric Neurology Turku University Hospital Turku Finland; ^12^ Neurocenter, Neurology Oulu University Hospital Oulu Finland; ^13^ Research Unit of Clinical Medicine and Medical Research Center Oulu University Hospital and University of Oulu Oulu Finland; ^14^ Department of Children and Adolescents, Division of Pediatric Neurology Oulu University Hospital Oulu Finland; ^15^ Finnish Epilepsy Association Finland

**Keywords:** care continuum, care model, epilepsy management, healthcare services, integrated care pathway, rare and complex epilepsy

## Abstract

**Objective:**

Integrated care pathways are essential for consistent, effective epilepsy care, offering equal access and quality regardless of socioeconomic status. They must align with the WHO Global Action Plan on Epilepsy, ensuring best practices and cost‐effective management. We describe the Finnish national epilepsy care pathway, which includes multiple levels of care, from initial diagnosis to long‐term care for all types of epilepsy, with a specific focus on rare and complex cases integrated with the European Reference Network (ERN) for Rare and Complex Epilepsies EpiCARE.

**Methods:**

In 2017, the Finnish government nominated Kuopio University Hospital to coordinate diagnostics and care for severe epilepsy in Finland. A national multidisciplinary consensus panel, including specialists from both adult and pediatric neurology departments across all five Finnish university hospitals and from the patient organization, was established. The resulting pathway was adopted into the current Finnish evidence‐based current care guidelines for epilepsy.

**Results:**

The Finnish epilepsy care pathway focuses on timely referrals, continuity of care and enhanced communication between healthcare providers at different levels of care. Patient involvement is assured with an individualized digital application offering secure online messaging, a seizure calendar, and remote visits. The pathway enhances virtual consultations and includes regular national diagnostic multidisciplinary meetings for severe epilepsies before selected cases are consulted in ERN EpiCARE meetings.

**Significance:**

This Finnish model for epilepsy care provides a streamlined, multidisciplinary approach to diagnosis and treatment and combines modern digital tools, data sharing, and peer support. This pathway can serve to model how integrated healthcare systems can effectively manage complex conditions.

**Plain Language Summary:**

We describe the Finnish national epilepsy care pathway, which includes multiple levels of care, from initial diagnosis to long‐term care for all types of epilepsy, with a specific focus on rare and complex cases integrated with the European Reference Network (ERN) for Rare and Complex Epilepsies EpiCARE. Finnish model for epilepsy care provides a streamlined, multidisciplinary approach to diagnosis and long‐term treatment of epilepsy. The pathway enhances virtual consultations and includes national and European‐level diagnostic multidisciplinary meetings for severe epilepsies. To improve outcomes, we emphasize the use of modern digital tools, data sharing, and peer support.

**Key points**

1. The Finnish model for epilepsy care provides a streamlined, multidisciplinary approach to diagnosis and treatment of epilepsy.
2. The model defines four levels of epilepsy care nationally and 5th level for European‐level reference network.
3. The pathway enhances virtual consultations and includes national and European‐level diagnostic multidisciplinary meetings for severe epilepsies.
4. The care pathway combines modern digital tools, data sharing, and peer support.

## 1 INTRODUCTION

Integrated care pathways (ICPs) can help ensure that patients receive timely and appropriate care, regardless of their place of residence or socioeconomic status.^1^ People‐centred, evidence‐based pathways are one of the country‐level goals of the WHO Intersectional Global Action Plan on Epilepsy and Other Neurological Disorders (WHO IGAP)^2^ and would improve equal access to and quality of care by recommending best practices corresponding to various stages of the condition. Care pathways utilize standardized procedures and recommendations for treatment to ensure consistency and uniformity in patient care. By providing a set of guidelines, ICPs can reduce variations in care, minimize errors, and promote better patient outcomes. They also provide a cost‐effective approach to medical management by minimizing the need for unnecessary tests and procedures. Therefore, ICPs help healthcare professionals (HCPs) provide optimal care for patients at different stages of treatment.

We describe the Finnish national epilepsy care pathway, which integrates multiple levels of care from diagnosis to long‐term care for all epilepsies, including complex and rare epilepsies integrated with the European Reference Network (ERN) for Rare and Complex Epilepsies (EpiCARE).^3^ It efficiently combines digital tools, data sharing, and peer support. This pathway, created by a government‐assigned consensus panel, demonstrates how comprehensive and integrated healthcare systems can be effectively used to manage complex conditions, such as epilepsy, serving as a model for other countries seeking to improve their epilepsy care standards.

## 2 METHODOLOGY

In 2017, the Finnish government decreed the division of labor in specialized healthcare.^4^ To improve healthcare and coordination, each of the five Finnish university hospitals was assigned responsibility for a specific area: cancer, autoimmune inflammatory rheumatic disease, sleep disturbances, health technology assessment, and severe epilepsy. Kuopio University Hospital (KUH) was tasked with coordinating diagnostics and care for severe epilepsy. A national Severe Epilepsy Coordination Group (SECG) was formed. The SECG included specialist representatives from both adult and pediatric neurology departments across all five Finnish university hospitals (20 specialists) and from the Finnish Epilepsy Association (FEA) as the patient organization (2 representatives). Finland is a geographically large country with a population of 5.6 million inhabitants. It is divided into 21 well‐being services counties, which provide comprehensive and reimbursed primary and specialized care. Every well‐being services county belongs to one collaborative area.^4^ There are a total of five collaborative areas, each with one university hospital promoting regional coordination. Therefore, the SECG represented all the well‐being services counties through their collaborative areas and had also discussed the care pathway with specialists in each collaborative area.

Using virtual consensus development methodology,^5^ the SECG (1) reviewed the literature and group members' experiences to identify key management gaps and (2) convened regularly through remote monthly meetings to make recommendations of the care pathway composition and determine the level of consensus. Five databases (PubMed, Embase, Scopus, Cochrane, and Google Scholar) were searched. Keyword search terms were based on four key concepts: epilepsy, care pathway, care continuum, healthcare, and integration. The resulting pathway was then adopted into the current Finnish evidence‐based care guidelines for epilepsy treatment in both children^6^ and adults^7^ as a consensus recommendation.

Finland's healthcare and social welfare system, including the epilepsy care pathway, is founded on public healthcare and social welfare services supported by government funds. Depending on the service health and social services are free of charge, or there is a client charge. The client charges have an upper limit per calendar year (in 2024 EUR 762) beyond which clients do not have to pay charges.

## 3 RESULTS

**FIGURE 1:**
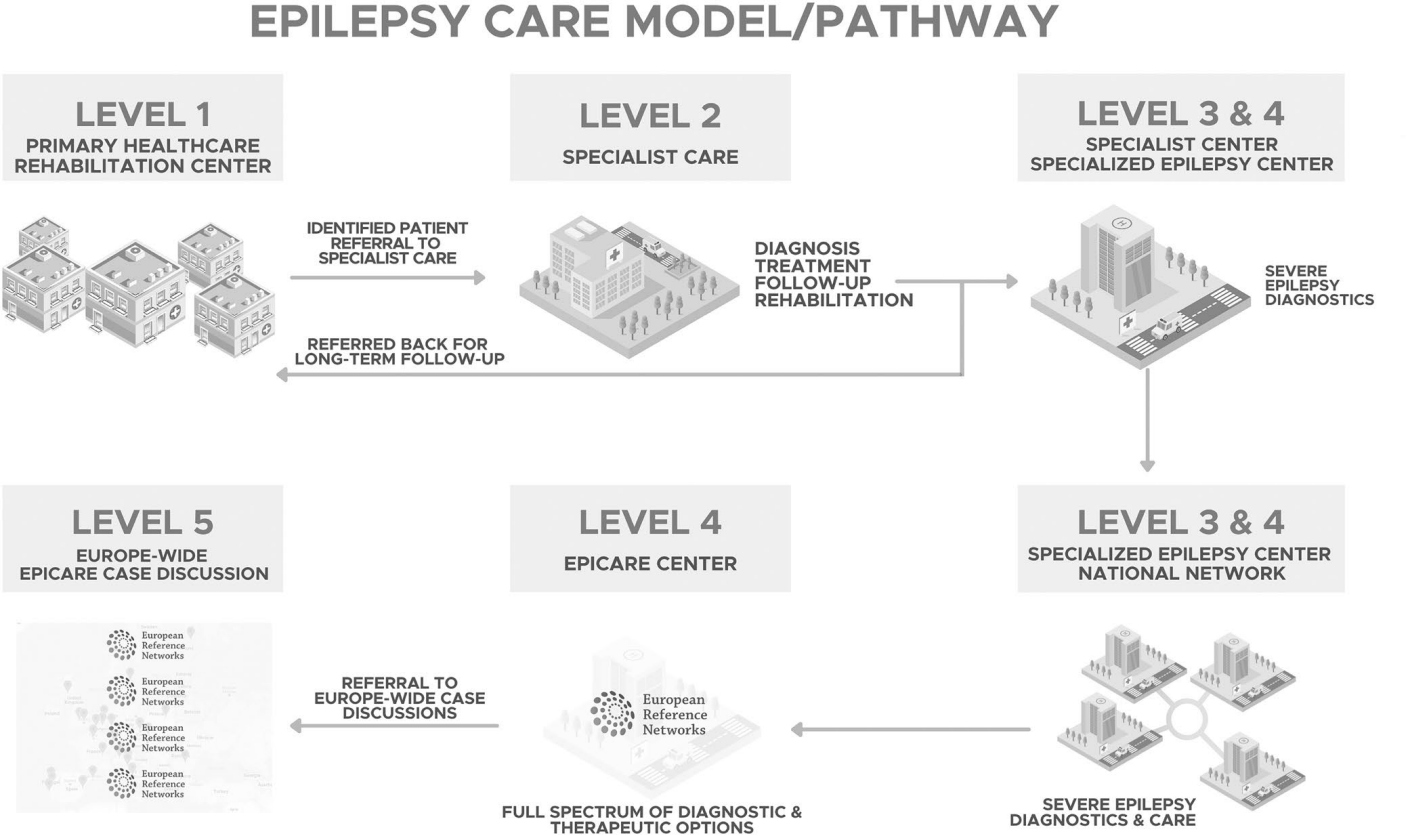
The diagram illustrates the multitiered structure of the Epilepsy Care Pathway, which consists of five levels of healthcare delivery. Level 1 is the Primary Healthcare, where initial patient identification and referral to specialist care occur. Level 2 represents Specialist Care facilities for diagnosis, treatment, follow‐up, and rehabilitation. Levels 3 and 4 are Specialist Centers and Specialized Epilepsy Centers, respectively, responsible for severe epilepsy diagnostics and care within a national network. The highest tier, Level 5, is the Europe‐wide EPICARE Case Discussion platform, facilitating cross‐border consultation and collaboration on complex cases within the European Reference Networks. Arrows indicate the referral pathways and the continuum of care across different levels, highlighting the system's integrated and patient‐centred approach.

The SECG defined five different levels of care, as well as the criteria for severe epilepsy and for referring patients to a more specialized level of care (Figure 1). The model covers both children and adolescents (under 16 years) and adults (16 years and older), as well as the transition from pediatric to adult care.

## 4 LEVEL 1: PRIMARY HEALTHCARE (NON‐SPECIALIST CARE)

At the primary healthcare level, the focus is on identifying the possibility of seizures and epilepsy. Patients with paroxysmal symptoms are assessed by a primary care physician after self‐referral. When seizures occur and epilepsy is suspected, a referral to a specialist for diagnostic examinations at Level 2 is issued by a general practitioner at the health center or an outpatient clinic for individuals with intellectual disabilities, or by an occupational health physician. The electronical referral includes a detailed description of the patient's seizure(s) and earlier medical history. Diagnostic testing is usually not performed at level 1. While waiting for the consultation, caregivers may be asked to record seizure(s) using their smartphones to aid in the diagnostic process.

Once an adult patient achieves seizure‐free status, care of comorbidities and vocational rehabilitation measures at Level 2 are planned, they return to Level 1 for follow‐up. If seizures start again, new adverse effects emerge or other problems arise, the patient is referred to Level 2 for consultation.

## 5 LEVEL 2: NEUROLOGY SPECIALIST CARE

At Level 2, specialist health services are provided by neurology and pediatric neurology units in central and university hospitals in each of the 21 well‐being county areas or for adults at private outpatient clinics. The diagnosis of epilepsy is made by a specialist after referral from Level 1 or from the emergency clinic of Level 2 if a patient needs emergency evaluation of the first seizure(s). Diagnostic processes include an evaluation of seizure type, syndrome, etiology, and possible comorbidities, as well as developmental support, educational, and vocational rehabilitation plans, driving ability and pregnancy planning and monitoring. The patient is given information about the diagnosis and treatment options, and the care plan is made concurrently with the patient after shared decision‐making.

Adult patients receive Level 2 care until they achieve seizure freedom. This also includes successfully implementing measures for their vocational rehabilitation, managing any existing comorbidities and ensuring a safe pregnancy, if applicable. Patients requiring hospice care or care during the palliative phases of treatment can also receive Level 2 care through remote consultation services. Pediatric patients receive specialist care throughout their childhood. The transition of care from pediatrics to adult Level 2 care usually takes place between the age of 16–18 years and needs to be planned with the family. Children with major learning disabilities can sometimes be referred for epilepsy follow‐up to Level 1 disability clinics, where they are cared for comprehensively throughout their lifespans.

## 6 LEVEL 3: EPILEPSY SPECIALIST CARE

The third level of the care pathway is the pediatric or adult neurology epilepsy units of university hospitals, which should be consulted remotely or by physical referral without delay in cases diagnosed with severe epilepsy. There are no private facilities currently offering diagnostic services for severe epilepsy in Finland. Severe epilepsy is defined as a state in which the patient, despite appropriate antiseizure medication (ASM), still experiences disabling symptoms that interfere with daily life. These symptoms can be seizures as well as adverse effects, cognitive problems, or developmental delays in children. The university hospitals are equipped with a multidisciplinary team (MDT) specializing in epilepsy, offering services such as video EEG, advanced imaging and genetic consultations. Level 3 care also encompasses re‐evaluations of a diagnosis, further etiological investigations, and detailed treatment planning, including coordination of dietary or orphan drug treatments. Stimulators, such as vagal nerve stimulators (VNSs), and in some centers, deep brain stimulators (DBSs), are implanted at university hospitals after presurgical evaluation at Level 4.

MDT epilepsy meetings are held regularly at university hospitals to discuss severe epilepsy cases. These meetings are attended by neurologists, neurophysiologists, neuropsychologists, neuroradiologists, and other specialists. The goal is to ensure that patients are treated with a comprehensive care approach. The process involves presenting a patient's epilepsy history, EEG and imaging findings, cognitive findings, comorbidities, and social situation, leading to a consensus regarding diagnosis and potential treatment options. Separate, ad hoc MDT meetings are also carried out for super refractory status epilepticus and other emergency cases as necessary.

## 7 LEVEL 4: SPECIALIZED EPILEPSY CENTERS

Level 4 encompasses presurgical assessment, intracranial recordings, epilepsy surgery, stimulator implantation (VNS and DBS), genetic consultations, and more complex epilepsy treatments. In Finland, there are two Level 4 centers: Kuopio Epilepsy Center at KUH and Epilepsia Helsinki at Helsinki University Hospital (HUS), where invasive recordings and epilepsy surgery have been centralized.

In addition to diagnostic MDT meetings, presurgical MDT meetings are held weekly. Level 3 HCPs participate regularly to level 4 MDTs, when their patients are discussed. Every 2 months, KUH and HUS also have presurgical MDT meetings and lead the national remote MDT meetings for severe epilepsy cases among the five university hospital MDTs. These national meetings are open to referring physicians wishing to attend on behalf of their patients.

## 8 LEVEL 5: EUROPEAN REFERENCE NETWORK (ERN) FOR RARE AND COMPLEX EPILEPSIES (EpiCARE)

Unresolved cases from the national severe case meetings or KUH/HUS presurgical MDT meetings may be presented to the ERN EpiCARE MDTs for consultations. From 2017 to 2021, KUH was the only ERN EpiCARE member from Finland, but in 2022, the HUS‐Oulu consortium was also selected as a member. Currently, the five university hospitals and their collaborative areas are connected to the ERN EpiCARE's activities via the SECG's activities and national MDT meetings with the aim of acting as a national consortium.

### 8.1 Digital tools used within the pathway

Health village^8^ is a public online service developed by Finnish university hospitals. Health village's digital care pathways complement traditional healthcare and appointments. Upon the initiation of a personal patient account (digital care pathway), patient‐specific data, such as seizure type, syndrome and etiology, are entered into the ‘My Epilepsy’ part of the account by HCPs. This process aims to ensure that a patient is provided with a comprehensive personalized understanding of their own condition. The symptom digital diary is a central feature that facilitates patients' chronological logging of their seizure events. Patients use the seizure calendar via their personal computer or mobile app and the data are integrated with their digital care pathway, providing HCPs with real‐time updates on a patient's condition, when HCPs log in the system during the visit or after the patient has sent a message via the digital care pathway. The messaging interface within the digital care pathway allows patients to initiate communication with HCPs at their convenience, allowing for timely and efficient clinical interactions with a clinic instead of routine contacts. Epilepsy nurses in each hospital answer the messages within 1–2 working days, after consulting a physician if necessary. Remote visits enable more detailed investigations and long‐term follow‐up after a diagnostic evaluation has been completed. Remote visits can also be an avenue for information dissemination and patient education.

### 8.2 Information sharing and databases

The epilepsy registry is a digital application integrated into hospitals' medical records.^9^, ^10^ The SECG agreed that all hospitals must include at least the following essential information in their respective registries: a patient's diagnosis, seizures, etiology of epilepsy, medications, and possible other treatments. The epilepsy registry also includes historical information on earlier seizures, changes in ASM and causes for stopping specific medications. Through an epilepsy registry, a neurologist can access an informative summary of a patient's epilepsy history and the effects of each therapy without consulting a patient's medical records. Specialists and epilepsy nurses enter the registry data usually during or immediately after the visits. HCPs can access the data for quality management. When the necessary research approvals and patient consent are appropriately in place, these data can also serve as significant resources for both national and international research. Examples of such uses include contributing to individual research projects using RedCAP Database^11^ such as ERN EpiCARE and other research on rare epilepsies.

In Finland, there is also a public service called Kanta that provides digital healthcare services.^12^ From Kanta, HCPs can view information from different healthcare units, while citizens can see their own health data, including electronic prescriptions, copies of their medical records of physician visits and laboratory and other test results. Furthermore, within MyKanta, it is possible to request prescription renewals, manage restrictions and agreements on personal data and confidentiality, and express intent on potential organ donation and end‐of‐life treatment.

## 9 COLLABORATION WITH THE NON‐PROFIT PATIENT ORGANIZATION AND PEER SUPPORT

Peer support is a vital component of the Finnish model of epilepsy care. The patient organization FEA and its local associations offer peer support at all levels, ensuring that individuals with epilepsy have access to emotional and practical support from others who have experienced similar challenges. In the initial stage, the focus is on epilepsy diagnosis and treatment, with HCPs (treating physicians and nurses) responsible for providing oral and written information and support. This stage is crucial in ensuring patients understand what kind of epilepsy they have, how to manage symptoms, and how to adhere to medication regimes. General information about epilepsy is provided on the health village's website,^8^ and information about self‐management and peer support can be found on the FEA's website^13^ Level 2 HCPs also arrange hybrid patient education days, to which recently diagnosed patients and their family members, as well as representatives of the FEA, are invited. In addition, at Levels 3 and 4, the FEA and HCPs arrange specific meetings regarding different rare and complex epilepsy diagnoses or specific treatments, such as VNS and epilepsy surgery.

After the initial phase, the role of the FEA, as well as its professionals, trained peer support volunteers, and local epilepsy associations, increases as a provider of information and support. The FEA, in collaboration with HCPs, organizes a variety of in‐person and online events, such as epilepsy courses, peer groups, and training, to share information and enable peer support. The volunteers are important experts, mediators, and exchangers of authentic experiences of daily life with epilepsy.

## 10 DISCUSSION

ICPs are designed to provide a systematic and multidisciplinary framework for managing patient care across different stages of chronic disease. Research indicates that well‐crafted ICPs can significantly improve the quality and consistency of care for epilepsy patients.^1^ Failure of services to meet current guidance leads to worse patient outcomes and additional health service costs.^14^, ^15^ The Finnish national epilepsy care pathway is an all‐encompassing system that integrates various stages of evidence‐based care, from the initial diagnosis to the development of long‐term treatment plans. It also seamlessly aligns the diagnostics and care practices for rare and complex epilepsies with the activities of ERN EpiCARE, highlighting the pathway's comprehensive and adaptive nature and aiming to close existing treatment gaps. Connecting our clinical registries after acquiring appropriate approvals and consent with the EpiCARE research database ensures that the management of epilepsy continues to align with the latest scientific advancements and discoveries.

The Finnish national epilepsy care pathway fulfills the goals set out by the WHO IGAP^2^ requiring health pathways to be evidence‐based and inclusive in terms of age, socioeconomic status, origin and orientation, coordinated care and social support, as well as optimized through the use of digital healthcare and tele‐consultations. Recent data show that the provision of epilepsy care across Europe is still quite variable^16^ Although the number of neurologists and pediatric neurologists per capita is relatively high in Finland,^17^, ^18^ remarkable regional variation exists. Finland's establishment of a national epilepsy care pathway can be extrapolated to other countries. Recently, also the National Neurosciences Advisory Group has overseen the development of an optimal care pathway for epilepsy for adults in UK.^19^ Both Finnish and UK processes emphasize the importance of national or regional cooperative planning, specialist networking, virtual consultations, structured virtual MDT meetings, data sharing, registers, and the rational use of digital tools for continuous improvements in patient care. Recently, published review of integrated care models for epilepsy revealed no other comprehensive program but identified 12 programs comprising various combinations of integrated care components.^20^

Early correct diagnoses and effective treatments improve the long‐term outcomes and quality of life of patients with epilepsy and are cost‐effective.^14^ Numerous factors may pose obstacles to the timely diagnosis of epilepsy^21^, ^22^ and represent opportunities for intervention. Increasing both public awareness and physicians' knowledge, particularly among non‐neurologists, about the range of seizure types and the impact of epilepsy are clearly areas that together with easy and equal access to diagnostic pathways, can improve patient outcomes, such as quality of life. Care pathways set standard procedures and recommendations for treatment to ensure consistency and uniformity in patient care. By integrating the Finnish epilepsy care pathway into current evidence‐based care guidelines, variations in care can be reduced and errors minimized.

Different criteria have been used to define drug‐resistant epilepsy for various purposes. The International League Against Epilepsy (ILAE) task force published a consensus definition of drug resistance. Its primary goal was to improve patient care and further clinical research. The ILAE defines drug‐resistant epilepsy as the failure of adequate trials of two tolerated and appropriately chosen and used ASM schedules (as monotherapy or in combination) to achieve sustained seizure freedom.^23^ Our SECG and Finnish guidelines discuss the concept of severe epilepsy and define it as a state in which a patient, despite appropriate drug treatment, still experiences disabling symptoms that interfere with daily life. These symptoms can be seizures, as well as adverse effects, cognitive problems or developmental delays in children. For the sake of guiding the timing of referrals and practical interventions in a patient's care pathway, adopting a broader interpretation of severe epilepsy including non‐seizure epilepsy symptoms has proven to be more useful.

Specialist networking and virtual consultations have been key elements in enabling more effective patient care in Finland. MDT meetings are crucial for optimal and consistent patient management, providing a structured, collaborative approach essential for informed decision‐making, learning and continuous improvement in treating complex conditions such as epilepsy.^24^ MDT meetings are hosted mostly virtually at Levels 2, 3, and 4 and between levels. Moreover, presurgical MDT meetings are held between the two Level 4 centers, and national MDTs for severe epilepsy cases among all five university hospitals every 2 months. Our Level 3 and 4 centers are able to provide the services that have been recommended by recent guidelines for specialized epilepsy centers in the USA.^25^ Selected cases are presented to the European‐level ERN EpiCARE MDTs for consultation (Level 5).

The care pathway, especially the digital care pathway, which provides an easy and secure way of contacting healthcare personnel, will help patients with epilepsy manage their condition more effectively and thus reduce costs by preventing unnecessary routine clinical visits and the need for emergency services.

When patient data are shared seamlessly between healthcare units, professionals have access to up‐to‐date information when treating patients. The Kanta service plays an invaluable role in increasing patient safety and healthcare standards. Furthermore, both the digital care pathway and the MyKanta service empower patients, typically making them feel more informed and facilitating better communication with HCPs.

Peer support is an integral part of the Finnish epilepsy care pathway at all levels, and close collaboration between HCPs and the FEA is essential. At the time of diagnosis, patients and their families need information and support from HCPs. In long‐term care, the focus shifts to training for self‐management and empowerment, as well as peer support. The HCP's role at this stage should predominantly focus on the medical management of epilepsy. By empowering patients in their self‐management and peer‐support possibilities, not only are HCPs' resources freed up for medical care, but overall costs are also effectively reduced.

The implementation of the Finnish national epilepsy care pathway started with the inclusion of the pathway in national and local care guidelines. Several articles on the pathway have also been published in *Epilepsia* magazine by the FEA^26^ and social media channels to inform patients.^13^ The SECG collects and analyses annually data on video EEG investigations, MDT meetings, surgical procedures, and the use of rare epilepsy treatments. The SECG will continue to survey physicians at different levels of the pathway to map the possible gaps in implementation and delays in diagnostics and care.

## 11 CONCLUSION

The Finnish epilepsy care pathway could serve as a template for other healthcare systems looking for a benchmark to improve their epilepsy care.

## AUTHOR CONTRIBUTIONS

*Conceptualization*: All authors. *Writing*‐*original draft* (lead): Reetta Kälviäinen and Zakarya Hadj‐Allal. *Writing*–*review and editing*: Jarkko Kirjavainen, Reina Roivainen, Tarja Linnankivi, Jukka Peltola, Kai Eriksson, Salla Lamusuo, Tuire Lähdesmäki, Johanna Annunen, Päivi Vieira, Virpi Tarkiainen, Leena Jutila, Anni Saarela, Leena Kämppi, Liisa Metsähonkala, Eija Gaily, Niina Lähde, Jaana Antinmaa, Sini Erme, Anna‐Leena Pirttisalo, Jari Virolainen, Milla Ylijoki, Laura Kela, Jonna Komulainen‐Ebrahim and Paula Sorjonen. Each author contributed significantly to the work, ensuring the accuracy and integrity of the research. All authors have reviewed and agreed on the final version of the manuscript and have agreed to be accountable for all aspects of the work, particularly those related to the accuracy and integrity of any part of the work.

## FUNDING INFORMATION

This research received a grant from the Finnish National Agency for Education (EDUFI), Finland.

## CONFLICT OF INTEREST STATEMENT

Reetta Kälviäinen: Grants from the Academy of Finland, Jane and Aatos Erkko Foundation, Saastamoinen Foundation, and Vaajasalo Foundation; honoraria from Eisai, Omamedical, Orion, Sandoz, Sanofi, and UCB; and honoraria for membership on the advisory boards of and consultation from Angelini Pharma, Eisai, Marinus Pharmaceuticals, Orion and UCB. Zakarya Hadj‐Allal: Honoraria from the U.S. Department of State, the UN's International Organization for Migration, the Association for Medical Education in Europe, ReInnovate Group, Orbital Learning Canada, and Medi Connection Oy, a grant from the Finnish National Agency for Education (EDUFI), Finland. Jarkko Kirjavainen: Travel support and expert fees from Biogen and Roche. Reina Roivainen: Personal fees from Angelini, Eisai, and Jazz Pharmaceuticals. Jukka Peltola: Clinical trials grants for Eisai, UCB, and Bial; research grants from Angelini Pharma, Eisai, Jazz Phara, Medtronic, UCB, and LivaNova; speaker's honoraria from LivaNova, Angelini Pharma, Eisai, Jazz Pharma, Medtronic, Orion Pharma and UCB; travel support from LivaNova, Eisai, Medtronic, and UCB; and on the advisory boards of LivaNova, Angelini Pharma, Jazz Pharma, Eisai, Medtronic, and UCB. Salla Lamusuo: Personal fees from Finnish Epilepsy Association, Eisai, Orion Pharma, and UCB. Tuire Lähdesmäki: Personal fees from Orion Pharma, Eisai, UCB, and Jazz Pharmaceuticals; research grants from the Finnish Government Research Funding, Finnish Pediatric Research Foundation, and Päivikki and Sakari Sohlberg Foundation. Johanna Annunen: Personal fees from Abbvie, Allergan, Angelini Pharma, Boston Scientific, Eisai, Genzyme, Jazz Pharmaceuticals, Orion Pharma, Roche, Sanofi and TEVA and has been involved in expert groups or lecturing for Allergan, Boston Scientific, Orion Pharma, Roche, Sanofi and TEVA. Päivi Vieira: Received personal fees from Eisai, Pfizer, Roche, and UCB. Virpi Tarkiainen: Grants for the FEA from Angelini Pharma, Jazz Pharmaceuticals, Nutricia Medical, OmaMedical, Orion Pharma, and UCB. Leena Jutila: Personal fees from Angelini Pharma, Eisai, UCB, and Novartis. Anni Saarela: Personal fees from UCB, Eisai, Pfizer, and Angelini Pharma, along with a grant from the Arvo and Lea Ylppö foundation. Leena Kämppi: Honoraria from UCB, Merck, and Eisai, travel support from UCB and Angelini Pharma, and research grants from the Finnish Cultural Foundation, Michael Foundation, Finnish Neurology Association, Academy of Finland, and HUS Neurocenter. Liisa Metsähonkala: Personal grants from Angelini Pharma, Takeda, Novartis, UCB, Eisai, Jazz Pharmaceuticals, Orion, and Marinus Pharmaceuticals and serves as the chair of the board of the FEA. Niina Lähde: Trial for UCB and honoraria from LivaNova (OmaMedical). Anna‐Leena Pirttisalo: Personal fees from Angelini Pharma. Laura Kela: Personal fees from TEVA. Jonna Komulainen‐Ebrahim: Personal fees from Jazz Pharmaceuticals. Paula Sorjonen: Grants for the FEA from Angelini Pharma, Jazz Pharmaceuticals, Nutricia Medical, OmaMedical, Orion Pharma, and UCB. Jarkko Kirjavainen: Travel support and expert fees from Biogen and Roche. Jukka Peltola: Clinical trials for Biohaven, Eisai, UCB, and Bial; research grants from Angelini Pharma, Eisai, Jazz Pharmaceuticals, Medtronic, UCB, and LivaNova; speaker's honoraria from LivaNova, Angelini Pharma, Eisai, Jazz Pharmaceuticals, Medtronic, Orion Pharma, and UCB; travel support from LivaNova, Eisai, Medtronic, and UCB; and on the advisory boards of LivaNova, Angelini Pharma, Jazz Pharmaceuticals, Eisai, Medtronic, and UCB. The following authors declare no conflicts of interest: Tarja Linnankivi, Kai Eriksson, Eija Gaily, Jaana Antinmaa, Sini Erme, Jari Virolainen, and Milla Ylijoki. We confirm that we have read the Journal's position on issues involved in ethical publication and affirm that thisreport is consistent with those guidelines.

## Data Availability

Data sharing is not applicable to this article as no new data were created or analyzed in this study.
